# Character Strengths Predict Subjective Well-Being, Psychological Well-Being, and Psychopathological Symptoms, Over and Above Functional Social Support

**DOI:** 10.3389/fpsyg.2021.661278

**Published:** 2021-09-21

**Authors:** Carolina M. Azañedo, Teresa Artola, Santiago Sastre, Jesús M. Alvarado

**Affiliations:** ^1^Department of Psychology, Villanueva University, Madrid, Spain; ^2^Department of Psychobiology & Behavioral Sciences Methods, Faculty of Psychology, Complutense University of Madrid, Madrid, Spain

**Keywords:** character strengths, social support, subjective well-being, psychological well-being, mental health, positive psychology

## Abstract

The increasing value of character strengths in the prediction of well-being and psychopathology, after the effects of functional social support and sociodemographic variables are accounted for, is examined. Participants were 1494 Spanish-speaking students between the ages of 18 and 68 (43.3% men and 56.7% women) who completed measures of character strengths, functional social support, subjective well-being, psychological well-being, and symptoms of psychopathology. Functional social support had predictive value in explaining the variability of each component of well-being and psychopathology. Regarding character strengths, theological strengths had the greatest predictive power for life satisfaction (β = 0.41), positive affect (β = 0.49), affect balance (β = 0.45), purpose in life (β = 0.60), self-acceptance (β = 0.50), environmental mastery (β = 0.47), and positive relations with others (β = 0.25). Emotional strengths made the strongest contribution to the variance explained (β = 0.41) of autonomy, and intellectual strengths were the strongest predictive variable for personal growth (β = 0.39). Strengths of restraint had the greatest predictive power for the global severity index of psychopathology (β = –0.27). Functional social support and character strengths have strong links to mental health. Positive interventions to develop these variables could contribute to enhance well-being and prevent psychological distress.

## Introduction

The Constitution of the World Health Organization defines good health as “a state of complete physical, social and mental well-being and not merely the absence of disease or infirmity” (World Health Organization [WHO], 1948). A positive concept of health derived from this definition, “emphasizing social and personal resources, as well as physical capacities” (World Health Organization [WHO], 1986). From this perspective, health is seen as a resource for everyday life. In line with this approach, the present study examined the role of character strengths and functional social support, as personal and social resources, on well-being and mental health.

Well-being research has been based on two philosophical approaches: the hedonic perspective, which defines well-being in terms of obtaining pleasure and avoiding pain; and the eudaimonic perspective, which formulates well-being in terms of the degree of full functioning of a person (Ryan and Deci, 2001). Concerning the hedonic psychological perspective, most research has used measures of subjective well-being (SWB) (Kahneman et al., 1999). Life satisfaction is considered the cognitive component of SWB and refers to global assessments of one’s life; positive affect and negative affect constitute the affective dimension of SWB and refer to the experiences of pleasant or unpleasant emotions, respectively (Ryan and Deci, 2001). Regarding the eudaimonic approach, Ryff (1989a) proposed a multidimensional model of psychological well-being (PWB) that comprised six components of positive functioning: self-acceptance (“positive evaluations of oneself and one’s past life”), positive relations with others (“the possession of quality relations with others”), autonomy (“a sense of self-determination”), environmental mastery (“the capacity to manage effectively one’s life and surrounding world”), purpose in life (“the belief that one’s life is purposeful and meaningful”), and personal growth (“a sense of continued growth and development as a person”) (Ryff and Keyes, 1995, p. 720). Therefore, whereas the SWB approach links well-being to life satisfaction and balance between positive and negative affect, the PWB approach formulates well-being in terms of perceived engagement with life’s existential challenges (Keyes et al., 2002).

Despite these differences in approach, most researchers affirm that well-being is best conceptualized as a complex phenomenon that includes dimensions of both the hedonic and eudaimonic perspectives (Ryan and Deci, 2001; Diener, 2009). Therefore, in this study, we used several hedonic and eudaimonic measures as indicators of well-being (Diener et al., 1985; Watson et al., 1988; Ryff, 1989b).

### The Role of Social Support on Well-Being and Mental Health

Social support is defined as “social resources that persons perceive to be available or that are actually provided to them by non-professionals in the context of both formal support groups and informal helping relationships” (Cohen et al., 2000, p. 4). Gottlieb and Bergen (2010) listed several support-related concepts such as social network, social integration, and functional support, which was defined as “the varied kinds of resources that flow through the network’s social ties” (p. 4). In particular, functional social support includes the following dimensions (Sherbourne and Stewart, 1991): (1) emotional support (demonstration of positive affect, empathy, and encouragement of expressions of feelings), (2) informational support (provision of advice, guidance, feedback, or information), (3) tangible support (offering of material resources or behavioral assistance), (4) positive social interaction (availability of others to have fun with us), and (5) affective support (demonstration of affection and love).

Social relationships have been associated with higher degrees of PWB and physical health at all ages (Walen and Lachman, 2000). Several studies have pointed to the significance of taking into account perceived social support in explaining the variance of SWB and PWB (e.g., Ferreira and Sherman, 2007; Khan and Husain, 2010; Yahaya et al., 2013; Skomorowsky, 2014). Previous research has shown that social support is linked to greater well-being and lower depressive symptoms (e.g., Limbert, 2004; Southwick et al., 2005; Guerette and Miller, 2011). Investigation has suggested that “people who have a strong psychological sense of support fare better in the face of adversity than those who are less sanguine about the support they can garner” (Gottlieb and Bergen, 2010, p. 512). Therefore, social support should be also considered as a factor that may buffer the impact of stress, adverse circumstances, or negative life events, such as a disease, on well-being and mental health (Henrich and Shahar, 2008; Beeble et al., 2009; Kutek et al., 2011; Costa et al., 2012). Specifically, perceived support is thought to buffer against stress by reducing the degree to which situations are perceived as threatening to well-being and enhancing the belief that necessary resources are available (Lakey and Cohen, 2000). Perceived social support also appears to increase the effectiveness of coping responses, which is associated with fewer symptoms of anxiety, depression, social withdrawal, and aggressive behavior (Calvete and Connor-Smith, 2006).

### The Role of Character Strengths on Well-Being and Mental Health

Character strengths have been described as morally valued positive traits, reflected in thoughts, feelings, and behaviors (Park et al., 2004). Signature strengths are those character strengths that are most typical of a person and that best characterize him or her (Peterson and Seligman, 2004). The *Values in Action* (VIA) classification (Peterson and Seligman, 2004) comprised 24 character strengths: social intelligence, perspective, creativity, bravery, humor, leadership, fairness, kindness, teamwork, modesty, forgiveness, self-regulation, prudence, persistence, open-mindedness, honesty, spirituality, gratitude, zest, hope, love, love of learning, curiosity, and appreciation of beauty and excellence. Several studies on the relationships among these 24 character strengths have shown that five-factorial solutions best fit the data (e.g., Ruch et al., 2010; Shryack et al., 2010; Littman-Ovadia and Lavy, 2012; Ruch et al., 2014). Higher-order strength factors have been generally labeled as follows: (1) emotional strengths (e.g., social intelligence, bravery), (2) interpersonal strengths (e.g., teamwork, kindness), (3) strengths of restraint (e.g., persistence, prudence), (4) theological strengths (e.g., spirituality, gratitude), and (5) intellectual strengths (e.g., curiosity, love of learning).

Research has revealed that character strengths have robust links to well-being in adults (e.g., Emmons and McCullough, 2003; Park et al., 2004; Peterson et al., 2007; Güsewell and Ruch, 2012; Buschor et al., 2013), youth and children (e.g., Park and Peterson, 2008; Gillham et al., 2011; Weber et al., 2013). Most of the evidence so far suggests that theological strengths (e.g., zest, love, hope, gratitude) are the character strengths most strongly connected to life satisfaction (e.g., Shimai et al., 2006; Gradisek, 2012; Proyer et al., 2013b). Regarding associations of character strengths with affective components of well-being, it has been found that the strengths of hope, curiosity, zest, love of learning, and perspective were the character strengths most strongly associated with positive affect, whereas the strengths of hope, curiosity, zest, love, and self-regulation showed significant (negative) correlations with negative affect (Littman-Ovadia and Lavy, 2012). With respect to the PWB, character strengths showed the strongest positive associations with the dimensions of environmental mastery, purpose in life and self-acceptance (Leontopoulou and Triliva, 2012).

In line with these findings, character strengths use has been shown to be an important predictor variable of well-being (e.g., Proctor et al., 2011). The use of signature strengths increases happiness and decreases depression (e.g., Seligman et al., 2005; Gander et al., 2013; Proyer et al., 2015). Strength-based interventions have been found to be effective in enhancing positive emotion (e.g., Seligman et al., 2006; Drozd et al., 2014) and life satisfaction (e.g., Rust et al., 2009; Duan et al., 2013), or improving levels of hopelessness and optimism (e.g., Huffman et al., 2014). In particular, character strengths may buffer from the negative effects of cognitive vulnerabilities (e.g., perfectionism, self-criticism, or excessive need for approval) that can lead to psychological ill-being, such as depression symptoms (Huta and Hawley, 2010). Additionally, greater reinforcement of character strengths has been linked to health behaviors (Proyer et al., 2013a), recovery from physical illness or psychological disorder (Peterson et al., 2006), and posttraumatic growth (Peterson et al., 2008).

### The Present Study

This study focused on the role of functional social support and character strengths on well-being and mental health. Specifically, the aim was to explore the amount of variance explained by perceived functional social support and each of the five higher-order strength factors (i.e., emotional strengths, interpersonal strengths, strengths of restraint, theological strengths, and intellectual strengths) in the following criterion variables: (a) SWB (i.e., life satisfaction, positive affect, negative affect, and affect balance); (b) PWB (i.e., self-acceptance, positive relations with others, autonomy, environmental mastery, purpose in life, and personal growth); and (c) symptoms of psychopathology or psychological disorders (i.e., somatization, obsessive-compulsive disorder, interpersonal sensitivity, depression, anxiety, hostility, phobic anxiety, paranoid ideation, and psychoticism). According to previous research, functional social support was expected to contribute significantly to explaining the variance of the components of SWB and PWB as well as psychopathological symptoms measured in this study. It was also hypothesized that character strengths contribute significantly to increase the amount of variance in each criterion variable explained by perceived functional social support. In an attempt to expand our understanding of whether there are specific strength factors that are more relevant to predict well-being and mental health than others, the current study examined the contribution of each character strengths factor to the criterion variables, over and above functional social support.

## Materials and Methods

### Participants

The sample consisted of 1494 university students (847 women, 647 men) enrolled in a degree in psychology. Their mean age was 33.99 years (*SD* = 10.99; range 18–68 years). Participants were recruited from five Spanish universities, three of them public (National University of Distance Education, Complutense University of Madrid, and King Juan Carlos University), and two of them private (Camilo José Cela University and Villanueva University). These universities were selected because the authors worked or had some type of working relationship with them at the time this study was conducted. All the participating universities were in Madrid, except for the National University of Distance Education, which has centers throughout Spain.

The inclusion criteria for participation in the study were the following: (a) to be 18 years old or older, (b) to be a university student enrolled in a psychology degree, (c) to be enrolled in one of the five Spanish universities mentioned above, and (d) to give informed consent to participate in the study. Table 1 presents data on the sociodemographic characteristics of the sample.

**TABLE 1:** Sociodemographic characteristics of the sample.

		Women		Men		Total	
		*Fr*	*%*	*Fr*	*%*	*Fr*	*%*
Age	18–24	267	31.54	180	27.75	447	29.92
	25–34	241	28.43	173	26.79	414	27.71
	35–44	213	25.09	142	22.01	355	23.76
	45–54	108	12.78	127	19.62	235	15.73
	55–68	18	2.15	25	3.83	43	2.88
		847	100	647	100	1494	100
Marital status	Single	448	52.89	319	49.30	767	51.34
	Married	216	25.50	196	30.29	412	27.58
	Living as a couple without being married	126	14.88	92	14.22	218	14.59
	Divorced or separated	54	6.38	37	5.72	91	6.09
	Widowed	3	0.35	3	0.46	6	0.40
		847	100	647	100	1494	100

*Fr, frequency or number of participants in each group. %, percentage of participants in each group.*

### Procedure

**FIGURE 1:**
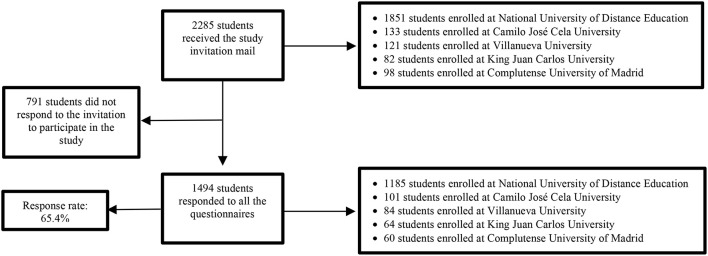
Sampling flowchart.

Participants provided personal sociodemographic data (i.e., age, gender, and marital status) and academic information (i.e., university enrolled). They also completed questionnaires to measure social support, character strengths, SWB and PWB, and symptoms of psychopathology. The questionnaires were administered online. Participants were sent a study invitation to their university email account. This e-mail provided the link to the study website from which the online questionnaires and informed consent were accessed. A total of 2285 students were invited to participate in the study, of which 1494 answered all the questionnaires. Data were collected between the 2016–2017 and 2019–2020 academic years. The sampling flowchart is shown in Figure 1.

### Instruments

The *Values in Action Inventory of Strengths* (VIA-IS; Peterson and Seligman, 2004; Peterson and Park, 2009) is a self-report questionnaire of 240 items in which the respondent reports the degree to which each statement applies to him/herself. To measure the 24 character strengths of the VIA Classification in the participants of this study, the Spanish adaptation of the VIA-IS (Azañedo et al., 2014) was used. The items are scored using a five-point Likert scale ranging from 1 (*very much unlike me*) to 5 (*very much like me*). The score for each of the 24 character strengths ranges from 10 to 50, with a higher score indicating a greater presence of the corresponding character strength. To obtain the 24 scores corresponding to the 24 character strengths, the mean of the items corresponding to each subscale was calculated. In order to identify the higher-order strength factors, we conducted a Parallel Analysis (Horn, 1965). Five components with eigenvalues that exceeded 1.00 and larger than the values generated randomly by Horn’s Parallel Analysis provided the best solution (the first ten eigenvalues were 10.39, 2.08, 1.65, 1.25, 1.12, 0.87, 0.79, 0.62, 0.59, and 0.53). The five-component solution accounted for 68.69% of the variance in the data (see Table 2). In line with previous studies (Peterson and Seligman, 2004; Park and Peterson, 2006; Ruch et al., 2010; Shryack et al., 2010; Littman-Ovadia and Lavy, 2012; Azañedo et al., 2014), we labeled our five factors with the following terms: emotional strengths, interpersonal strengths, strengths of restraint, theological strengths, and intellectual strengths. The first factor, *emotional strengths*, explained 18.29% of the variance, and was loaded by the strengths of social intelligence, perspective, creativity, bravery, humor, and leadership. The factor of *interpersonal strengths* explained about 17.36% of the variance, and included the strengths of fairness, kindness, teamwork, modesty, and forgiveness. The factor named *strengths of restraint* explained 11.89% of the variance, and comprised the strengths of self-regulation, prudence, persistence, open-mindedness, and honesty. The *theological strengths* factor explained 11.67% of the variance and was loaded by the strengths of spirituality, gratitude, zest, hope, and love. The factor representing *intellectual strengths* explained less variance (9.48%) and contained the character strengths of love of learning, appreciation of beauty and excellence, and curiosity. The character strengths included in our five factors were the same as those obtained by Azañedo et al. (2014) and loaded the same way. However, our five components were not identical to the five factors obtained by the other reports cited (i.e., Peterson and Seligman, 2004; Park and Peterson, 2006; Ruch et al., 2010; Shryack et al., 2010; Littman-Ovadia and Lavy, 2012). Thus, most of the groups of character strengths that composed each factor were similar to those of the previous models, but not exactly the same.

**TABLE 2:** Five-factor solution for the VIA-IS.

Strengths	Factor1	Factor2	Factor3	Factor4	Factor5	*h* ^2^
Social intelligence	**0.72**	0.27	0.19	0.20	0.09	0.68
Perspective	**0.72**	0.15	0.37	0.05	0.23	0.72
Creativity	**0.70**	–0.04	0.06	0.07	0.43	0.68
Bravery	**0.67**	0.11	0.24	0.25	0.14	0.60
Humor	**0.67**	0.34	–0.12	0.30	0.02	0.66
Leadership	**0.56**	0.53	0.17	0.17	0.09	0.66
Fairness	0.23	**0.77**	0.23	0.01	0.23	0.75
Kindness	0.32	**0.72**	0.05	0.31	0.09	0.72
Teamwork	0.30	**0.71**	0.12	0.30	–0.04	0.71
Modesty	–0.10	**0.70**	0.36	0.03	0.03	0.63
Forgiveness	0.07	**0.68**	0.16	0.24	0.24	0.60
Self-regulation	0.18	0.15	**0.74**	0.28	0.11	0.70
Prudence	0.06	0.46	**0.72**	–0.01	0.14	0.75
Persistence	0.28	0.08	**0.68**	0.44	0.07	0.75
Open-mindedness	0.51	0.24	**0.55**	–0.15	0.35	0.76
Honesty	0.41	0.45	**0.50**	0.13	0.05	0.63
Spirituality	0.04	0.15	0.16	**0.65**	0.14	0.49
Gratitude	0.20	0.46	0.10	**0.63**	0.31	0.75
Zest	0.50	0.14	0.22	**0.63**	0.23	0.76
Hope	0.48	0.16	0.30	**0.58**	0.20	0.73
Love	0.32	0.48	–0.08	**0.51**	0.05	0.60
Love of learning	0.15	0.06	0.26	0.09	**0.78**	0.71
Appreciation of beauty	0.16	0.29	–0.05	0.26	**0.72**	0.69
Curiosity	0.44	0.11	0.16	0.34	**0.63**	0.74
Explained variance (in%)	18.29	17.36	11.89	11.67	9.48	68.69

***Bold** indicates highest factor loadings of the subscales.*

*h^2^, communality.*

Five final scores were computed for each participant by averaging the character strengths scores assigned to each higher-order strength factor. These five scores (i.e., five higher-order character strengths factor scores) were used for the analyses. In the current sample, the five scales had satisfactory reliability (mean α = 0.83; mean corrected item-total correlations = 0.64). Specifically, Cronbach’s α was 0.86 for emotional strengths, 0.85 for interpersonal strengths, 0.84 for strengths of restraint, 0.82 for theological strengths, and 0.76 for intellectual strengths.

The *MOS Social Support Survey* (Sherbourne and Stewart, 1991) is a 20-item self-report that was developed for patients in the Medical Outcomes Study and measures the perception of social support. Item number 1 refers to the social network size and the remaining 19 items refers to the following dimensions of functional social support: emotional/informational support, tangible support, positive social interaction and affective support. Each of these 19 items is scored on a five-point scale that ranges from 1 (*never*) to 5 (*always*). One overall functional social support index was computed for each participant by averaging the scores of the four dimensions of functional social support. In this study we used the Spanish version validated by De la Revilla et al. (2005), which revealed internal consistency values, measured by Cronbach’s α, ranging from 0.86 (affective support) to 0.94 (emotional/informational support). In the present sample, the Cronbach’s α coefficients were 0.95 for emotional/informational support, 0.88 for tangible support, 0.90 for positive social interaction, and 0.86 for affective support.

The *Satisfaction with Life Scale* (SWLS; Diener et al., 1985) is a self-report questionnaire through which the respondent assesses his or her satisfaction with life in general. This instrument consists of five items or statements to which the participant responds using a 5-point scale ranging from 1 (*strongly disagree*) to 5 (*strongly agree*). The SWLS has shown adequate psychometric properties to measure this cognitive component of SWB (Pavot and Diener, 2008). The students who participated in this study completed the Spanish version of the SWLS (Atienza et al., 2000), which showed an internal consistency measured by Cronbach’s α coefficient of 0.84. The mean of the scores on the five items was calculated to obtain an overall life satisfaction score for each participant, where a higher score reflected higher degree of life satisfaction. In this study we obtained a Cronbach’s α coefficient of 0.88 and values over 0.60 for all corrected item-total correlations. In the study conducted by Vázquez et al. (2013) in a Spanish sample, the same psychometric results were obtained.

The Positive and Negative Affect Schedule (PANAS; Watson et al., 1988) is a self-report questionnaire composed of two subscales, one measuring Positive Affect (PA) and the other measuring Negative Affect (NA). Each subscale consists of 10 items, specifically 10 adjectives reflecting positive affect for the PA subscale (e.g., enthusiastic, inspired), and 10 adjectives describing negative affect for the NA subscale (e.g., irritable, fearful). Students rated each of the 20 adjectives in terms of how they had felt over the past few days using a five-point scale ranging from 1 (*very slightly or not at all*) to 5 (*extremely*). The mean of the items corresponding to each subscale was calculated separately, thus obtaining two scores for each participant (one score in PA and another in AN). A high score indicated a greater presence of the corresponding affective dimension. To calculate the affective balance score, the NA subscale score was subtracted from the PA subscale score. In previous studies with different samples (e.g., Terracciano et al., 2003; Crawford and Henry, 2004), adequate psychometric properties of the PANAS have been reported. For the Spanish version of this instrument validated by Sandín et al. (1999), a Cronbach’s α coefficient of 0.89 was obtained for the PA subscale and 0.91 for the NA subscale in men, and 0.87 for the PA subscale and 0.89 for the NA subscale in women. In the sample of this study, a Cronbach’s α coefficient of 0.92 was found for the PA subscale and 0.89 for the NA subscale.

The *Psychological Well-Being Scales* (PWBS; Ryff, 1989b) is a self-report comprising the following scales of PWB: self-acceptance, positive relations with others, autonomy, environmental mastery, purpose in life, and personal growth (Ryff and Keyes, 1995, p. 720). In this study, we used the 29-item version proposed by Díaz et al. (2006), in which each item was scored on a six-point scale from 1 (*totally disagree*) to 6 (*totally agree*). For this Spanish adaptation of the PWBS, Cronbach’s α coefficient values for the six scales ranged from 0.84 (self-acceptance) to 0.70 (autonomy and purpose in life). In the current study, we calculated the internal consistency coefficient (α) for each scale:0.83 for self-acceptance, 0.80 for positive relationships with others, 0.73 for autonomy, 0.73 for mastery of the environment, 0.84 for purpose in life, and 0.74 for personal growth. Scores on the six PWB dimensions were averaged to obtain an overall PWB index for each participant. A high score on the PWBS indicated a high presence of PWB.

The *Symptom Checklist-90-Revised* (SCL-90-R; Derogatis, 1994) is a 90-item self-report that assesses a wide range of symptoms of psychopathology. This instrument uses a five-point Likert scale of distress, which ranges from 0 (*not at all*) to 4 (*extremely*). The SCL-90-R evaluates nine psychological problems or symptom dimensions: somatization (e.g., distress arising from the perception of bodily dysfunctions, headaches, muscular pain and other somatic manifestations of anxiety), obsessive-compulsive symptoms (e.g., thoughts, actions and impulses experienced as involuntary, undesired and impossible to control), interpersonal sensitivity (e.g., feelings of inferiority and discomfort in interpersonal relationships), depression (e.g., feelings of hopelessness, loss of vital energy, dysphoric mood), anxiety (e.g., restlessness, nervousness, tension and panic attacks), hostility (e.g., thoughts, feelings, or actions characteristic of anger, such as aggression), phobic anxiety (e.g., persistent fear response to a specific person, place, object, or situation, which leads to avoidance or escape behavior), paranoid ideation (e.g., suspiciousness, grandiosity), and psychoticism (e.g., auditory hallucinations, transmission and thought control) In this study, we used the Spanish adaptation developed by González de Rivera, De las Cuevas, Rodríguez, and Rodríguez (see Derogatis, 2002), which shows good levels of internal consistency for all the scales (with Cronbach’s α ranging from 0.81 to 0.90). In the present sample, Cronbach’s α was 0.86 for somatization, 0.87 for obsessive-compulsive subscale, 0.85 for interpersonal sensitivity, 0.91 for depression, 0.86 for anxiety, 0.84 for hostility, 0.74 for phobic anxiety, 0.78 for paranoid ideation, and 0.78 for psychoticism. In the current study, we used the summary score named Global Severity Index (GSI) since it is the best indicator of distress severity and should be utilized when a single summary score is needed (Derogatis, 1994). The GSI is obtained by adding the scores corresponding to the 90 items that compose the questionnaire and then dividing the result of this sum by the total number of items.

### Data Analysis

The SPSS program (version 25) was applied to conduct the data analyses. As for the descriptive analysis of the data, frequencies and percentages were computed for the categorical variables, and minimum and maximum values, mean scores and standard deviations were calculated for the rest of the study variables. Correlation analyses were used to examine the relationships between study variables. Multiple linear hierarchical regression analyses were performed using as dependent variables components of SWB, dimensions of PWB, and psychological problems. In order to compare the predictive capacity of each of the five higher-order strength factors on well-being and mental health, we conducted five multiple regression analyses for each dependent variable. We controlled for sociodemographic characteristics of gender and age by entering them in the first and second blocks, respectively. Then we introduced the overall functional social support index in the third step. Finally, we entered one of the five higher-order strength factors (i.e., emotional strengths, interpersonal strengths, strengths of restraint, theological strengths, and intellectual strengths) in each regression analysis. Not entering the five strength scales simultaneously in the fourth step of each model was related to the possibility of multicollinearity among these strength scores. Selection of the predictor variables was performed with the level of significance *p* < 0.05.

## Results

### Descriptive and Correlation Analyses

The descriptive statistics of mean, standard deviation, and minimum and maximum value for the study variables are presented in Table 3. The response ranges corresponding to the different instruments used to measure the variables are also shown in Table 3 in order to facilitate the interpretation of the descriptive statistics. Strength scale means, on a potential 1–5 scale, range from 3.71 (theological strengths) through 3.95 (intellectual strengths). Standard deviations ranged from 0.46 (emotional and interpersonal strengths) through 0.52 (theological strengths). In the present sample, the descriptive results also revealed high levels of functional social support (*M* = 4.24, *SD* = 0.76). Table 3 also provides the partial correlation coefficients (adjusting the effect of gender and age) between the study variables. Because of the large number of correlations, we used a conservative *p* level of.001.

**TABLE 3:** Descriptive statistics, answer ranges and partial correlations (adjusting the effect of gender and age) between the variables (*N* = 1494).

Variable	*Answer range*	*Min*	*Max*	*M*	*SD*	Gender^(a)^	(1)	(2)	(3)	(4)	(5)	(6)	(7)	(8)	(9)	(10)	(11)	(12)	(13)	(14)	(15)	(16)	(17)	(18)	(19)	(20)	(21)	(22)	(23)	(24)	(25)	(26)	(27)
**(1) Age**	From 18	18	68	33.99	10.99	–0.16*	1.00																										
**(2) Functional social support**	From 1 to 5	1.00	5.00	4.24	0.76	0.12*	–0.01	1.00																									
**(3) Emotional strengths**	From 1 to 5	1.90	5.00	3.78	0.46	–0.08	–0.01	0.27*	1.00																								
**(4) Interpersonal strengths**	From 1 to 5	1.86	4.94	3.94	0.46	0.11*	0.11*	0.22*	0.56*	1.00																							
**(5) Strengths of restraint**	From 1 to 5	1.82	4.94	3.78	0.48	–0.03	0.16*	0.21*	0.64*	0.61*	1.00																						
**(6) Theological strengths**	From 1 to 5	1.72	5.00	3.71	0.52	0.07	0.11*	0.37*	0.71*	0.59*	0.59*	1.00																					
**(7) Intellectual strengths**	From 1 to 5	2.00	5.00	3.95	0.49	0.01	0.23*	0.20*	0.61*	0.42*	0.48*	0.59*	1.00																				
(8) Life satisfaction	From 1 to 5	1.00	5.00	3.58	0.84	0.01	0.08	0.48*	0.40*	0.28*	0.37*	0.53*	0.32*	1.00																			
(9) Positive affect	From 1 to 5	1.00	5.00	3.48	0.77	–0.13*	0.12*	0.33*	0.53*	0.26*	0.40*	0.56*	0.48*	0.52*	1.00																		
(10) Negative affect	From 1 to 5	1.00	5.00	1.95	0.75	0.02	–0.17*	–0.28*	–0.24*	–0.23*	–0.30*	–0.30*	–0.17*	–0.45*	–0.28*	1.00																	
(11) Affect balance	From –4 to 4	–2.80	4.00	1.53	1.23	–0.09*	0.18*	0.38*	0.49*	0.30*	0.44*	0.54*	0.41*	0.61*	0.81*	–0.79*	1.00																
(12) Self- acceptance	From 1 to 6	1.00	6.00	4.63	0.97	–0.02	0.08	0.39*	0.53*	0.30*	0.46*	0.60*	0.41*	0.74*	0.58*	–0.47*	0.66*	1.00															
(13) Positive relations with others	From 1 to 6	1.00	6.00	4.65	1.02	0.14*	–0.03	0.49*	0.35*	0.34*	0.25*	0.40*	0.24*	0.39*	0.33*	–0.27*	0.38*	0.47*	1.00														
(14) Autonomy	From 1 to 6	1.00	6.00	4.18	0.89	–0.07	0.10*	0.16*	0.42*	0.13*	0.31*	0.22*	0.24*	0.27*	0.27*	–0.34*	0.38*	0.44*	0.28*	1.00													
(15) Environmental mastery	From 1 to 6	1.00	6.00	4.48	0.89	–0.02	0.19*	0.41*	0.47*	0.31*	0.47*	0.58*	0.39*	0.67*	0.55*	–0.45*	0.63*	0.75*	0.48*	0.41*	1.00												
(16) Purpose in life	From 1 to 6	1.00	6.00	4.62	0.93	0.04	0.11*	0.36*	0.53*	0.34*	0.52*	0.66*	0.43*	0.64*	0.57*	–0.37*	0.59*	0.78*	0.42*	0.34*	0.75*	1.00											
(17) Personal growth	From 1 to 6	1.00	6.00	5.11	0.81	0.03	0.05	0.20*	0.41*	0.27*	0.32*	0.40*	0.40*	0.34*	0.39*	–0.20*	0.37*	0.55*	0.34*	0.33*	0.50*	0.53*	1.00										
(18) Overall psychological well-being index	From 1 to 6	1.00	5.97	4.61	0.69	0.03	0.11*	0.45*	0.60*	0.38*	0.51*	0.63*	0.46*	0.68*	0.59*	–0.47*	0.66*	0.88*	0.67*	0.61*	0.86*	0.84*	0.70*	1.00									
(19) Somatization	From 0 to 4	0.00	3.92	0.54	0.58	0.21*	–0.12*	–0.20*	–0.11*	–0.08	–0.19*	–0.17*	–0.09	–0.32*	–0.23*	0.49*	–0.45*	–0.31*	–0.21*	–0.15*	–0.31*	–0.25*	–0.14*	–0.30*	1.00								
(20) Obsessive–compulsive	From 0 to 4	0.00	3.60	0.82	0.73	0.11*	–0.20*	–0.33*	–0.30*	–0.19*	–0.37*	–0.32*	–0.23*	–0.50*	–0.44*	0.61*	–0.65*	–0.52*	–0.35*	–0.36*	–0.52*	–0.46*	–0.29*	–0.55*	0.59*	1.00							
(21) Interpersonal sensitivity	From 0 to 4	0.00	3.56	0.63	0.63	0.10*	–0.21*	–0.37*	–0.33*	–0.25*	–0.32*	–0.32*	–0.22*	–0.49*	–0.34*	0.59*	–0.57*	–0.54*	–0.44*	–0.44*	–0.48*	–0.42*	–0.28*	–0.58*	0.52*	0.70*	1.00						
(22) Depression	From 0 to 4	0.00	3.62	0.77	0.74	0.15*	–0.19*	–0.42*	–0.31*	–0.21*	–0.33*	–0.42*	–0.23*	–0.61*	–0.48*	0.69*	–0.73*	–0.61*	–0.41*	–0.32*	–0.60*	–0.52*	–0.29*	–0.61*	0.61*	0.79*	0.75*	1.00					
(23) Anxiety	From 0 to 4	0.00	3.50	0.57	0.60	0.14*	–0.23*	–0.27*	–0.20*	–0.16*	–0.27*	–0.24*	–0.15*	–0.43*	–0.29*	0.73*	–0.63*	–0.45*	–0.26*	–0.30*	–0.43*	–0.36*	–0.20*	–0.44*	0.68*	0.70*	0.67*	0.74*	1.00				
(24) Hostility	From 0 to 4	0.00	3.67	0.45	0.63	0.05	–0.21*	–0.25*	–0.18*	–0.25*	–0.26*	–0.21*	–0.17*	–0.38*	–0.27*	0.56*	–0.51*	–0.36*	–0.23*	–0.19*	–0.34*	–0.30*	–0.21*	–0.36*	0.47*	0.54*	0.56*	0.60*	0.58*	1.00			
(25) Phobic anxiety	From 0 to 4	0.00	3.29	0.20	0.39	0.07	–0.09*	–0.21*	–0.16*	–0.14*	–0.17*	–0.18*	–0.12*	–0.29*	–0.21*	0.44*	–0.40*	–0.32*	–0.24*	–0.20*	–0.29*	–0.27*	–0.15*	–0.33*	0.48*	0.50*	0.57*	0.51*	0.64*	0.36*	1.00		
(26) Paranoid ideation	From 0 to 4	0.00	4.00	0.67	0.69	0.00	–0.20*	–0.36*	–0.13*	–0.21*	–0.20*	–0.19*	–0.11*	–0.41*	–0.21*	0.49*	–0.44*	–0.35*	–0.38*	–0.22*	–0.38*	–0.30*	–0.17*	–0.40*	0.45*	0.61*	0.74*	0.66*	0.58*	0.56*	0.47*	1.00	
(27) Psychoticism	From 0 to 4	0.00	2.90	0.37	0.46	–0.01	–0.19*	–0.36*	–0.21*	–0.20*	–0.27*	–0.27*	–0.17*	–0.47*	–0.31*	0.54*	–0.53*	–0.47*	–0.39*	–0.30*	–0.45*	–0.38*	–0.26*	–0.50*	0.58*	0.70*	0.73*	0.74*	0.70*	0.53*	0.58*	0.69*	1.00
(28) Global severity index (GSI)	From 0 to 4	0.00	2.98	0.59	0.51	0.13*	–0.22*	–0.38*	–0.28*	–0.23*	–0.34*	–0.34*	–0.21*	–0.55*	–0.40*	0.72*	–0.70*	–0.56*	–0.40*	–0.35*	–0.54*	–0.46*	–0.29*	–0.57*	0.76*	0.87*	0.84*	0.91*	0.86*	0.69*	0.65*	0.76*	0.85*

*Answer range, answer range of the respective questionnaire; *Min*, minimum value; *Max*, maximum value; *M*, mean; *SD*, standard deviation; (a) 1- Men (43.3%), 2- Women (56.7%); predictor variables are marked in bold type.*

*p < 0.001.

Regarding relations between character strengths, all partial correlation coefficients were positive and greater than 0.40. The highest partial correlation was found between theological strengths and emotional strengths (*r* = 0.71). We found that all strength factors except emotional strengths were significantly and directly related to age. These correlations were generally small and oscillated between 0.11 (interpersonal and theological strengths) and 0.23 (intellectual strengths). The correlations with gender indicated higher functional social support scores and higher interpersonal strengths for women. The five strength factors correlated significantly with functional social support. Specifically, theological strengths were the strengths linked to higher functional social support level (*r* = 0.37).

Functional social support and all strength factors were significantly and directly related to life satisfaction, positive affect, and affect balance. In contrast, negative affect was significantly and negatively related to functional social support (*r* = –0.28) and the five strength scales, ranging from –0.17 (intellectual strengths) through –0.30 (strengths of restraint and theological strengths). We also found that older age correlated significantly with higher score for the PA subscale (*r* = 0.12), lower score for the NA subscale (*r* = –0.17), and higher score for affect balance (*r* = 0.18).

As shown in Table 3, functional social support was significantly related to dimensions of PWB. These significant correlations were between 0.16 (autonomy) and 0.49 (positive relations with others). All strength scales had significant positive correlations with every component of PWB. Specifically, theological strengths yielded the highest (positive) correlation with the overall PWB index (*r* = 0.63). Regarding correlations between gender and PWB, women were more likely to score higher on positive relations with others (*r* = 0.14). Older age correlated with higher scores for autonomy (*r* = 0.10), purpose in life (*r* = 0.11), and environmental mastery (*r* = 0.19).

Correlations between symptoms of psychopathology and functional social support were negative and significant, ranging from –0.20 (somatization) through –0.42 (depression). The correlations with gender indicated higher scores for women for the subscales of somatization (*r* = 0.21), obsessive-compulsive symptoms (*r* = 0.11), interpersonal sensitivity (*r* = 0.10), depression (*r* = 0.15), and anxiety (*r* = 0.14). We found that younger age correlated significantly with higher score for all symptom dimensions. These significant correlations were between –0.09 (phobic anxiety) and –0.23 (anxiety).

### Hierarchical Multivariate Regression Analyses

Table 4 shows the results of hierarchical multivariate regression analyses for the variables predicting the components of SWB, PWB, and symptoms of psychopathology. For positive affect and affect balance, both gender and age entered in the models at a significant level. For the components of life satisfaction and negative affect, only sociodemographic characteristic of age entered the final models at a significant level. Functional social support was found to be a variable with predictive value in explaining the variability of each dimension of SWB in the third step. Specifically, this predictor produced the highest increment of *R*^2^ for the dimension of life satisfaction (*R*^2^ change = 0.23), and the lowest significant increment of this value for the dimension of negative affect (*R*^2^ change = 0.08). Concerning character strengths as predictors of SWB, results showed that theological strengths had the greatest predictive power for life satisfaction (β = 0.41), positive affect (β = 0.49), and affect balance (β = 0.45). With regard to negative affect, the model composed of age, functional social support and strengths of restraint explained the highest percentage of variance for this dimension (*R*^2^ = 0.16).

**TABLE 4:** Predicting subjective well-being, psychological well-being, and symptoms of psychopathology.

**Order of entry**	**1st and 2nd blocks**		**3rd block**	**4th block**				
	**Sociodemographic characteristics**		**Functional social support**	**Character strengths**				
	**Gender^(a)^**	**Age**	**Overall support index**	**Emotional strengths**	**Interpersonal strengths**	**Strengths of restraint**	**Theological strengths**	**Intellectual strengths**
**Life satisfaction**								
*R*^2^ adj.	–	0.01	0.24	0.31	0.27	0.31	0.38	0.29
*R*^2^ change	–	0.01	0.23	0.08	0.03	0.08	0.14	0.05
β (*SE*)	–	0.08** (0.00)	0.48*** (0.03)	0.29*** (0.04)	0.18*** (0.04)	0.28*** (0.04)	0.41*** (0.04)	0.24*** (0.04)
**Positive affect**								
*R*^2^ adj.	0.02	0.03	0.13	0.33	0.16	0.24	0.33	0.30
*R*^2^ change	0.02	0.01	0.10	0.20	0.03	0.11	0.20	0.17
β (*SE*)	–0.14*** (0.04)	0.10*** (0.00)	0.32*** (0.03)	0.47*** (0.04)	0.19*** (0.04)	0.34*** (0.04)	0.49*** (0.04)	0.43*** (0.04)
**Negative affect**								
*R*^2^ adj.	–	0.03	0.10	0.14	0.13	0.16	0.15	0.12
*R*^2^ change	–	0.03	0.08	0.03	0.03	0.06	0.04	0.01
β (*SE*)	–	–0.16*** (0.00)	–0.28*** (0.03)	–0.19*** (0.04)	–0.18*** (0.04)	–0.25*** (0.04)	–0.23*** (0.04)	–0.12*** (0.04)
**Affect balance**								
*R*^2^ adj.	0.01	0.04	0.18	0.33	0.22	0.30	0.35	0.29
*R*^2^ change	0.01	0.03	0.14	0.16	0.05	0.13	0.17	0.11
β (*SE*)	–0.11*** (0.07)	0.16*** (0.00)	0.38*** (0.04)	0.41*** (0.06)	0.23*** (0.06)	0.37*** (0.06)	0.45*** (0.06)	0.35*** (0.06)
**Self-acceptance**								
*R*^2^ adj.	–	0.01	0.16	0.34	0.20	0.30	0.37	0.26
*R*^2^ change	–	0.01	0.15	0.19	0.04	0.14	0.21	0.10
β (*SE*)	–	0.09** (0.00)	0.39*** (0.03)	0.45*** (0.05)	0.21*** (0.05)	0.39*** (0.05)	0.50*** (0.04)	0.33*** (0.05)
**Positive relations with others**								
*R*^2^ adj.	0.02	–	0.25	0.30	0.31	0.27	0.30	0.27
*R*^2^ change	0.02	–	0.23	0.05	0.05	0.02	0.05	0.02
β (*SE*)	0.14*** (0.05)	–	0.49*** (0.03)	0.23*** (0.05)	0.24*** (0.05)	0.14*** (0.05)	0.25*** (0.05)	0.13*** (0.05)
**Autonomy**								
*R*^2^ adj.	–	0.01	0.03	0.19	0.04	0.11	0.06	0.08
*R*^2^ change	–	0.01	0.02	0.16	0.01	0.08	0.03	0.04
β (*SE*)	–	0.10*** (0.00)	0.15*** (0.03)	0.41*** (0.05)	0.09** (0.05)	0.30*** (0.05)	0.17*** (0.05)	0.22*** (0.05)
**Environmental mastery**								
*R*^2^ adj.	–	0.03	0.19	0.32	0.23	0.33	0.38	0.28
*R*^2^ change	–	0.03	0.16	0.13	0.04	0.14	0.19	0.09
β (*SE*)	–	0.18*** (0.00)	0.40*** (0.03)	0.37*** (0.04)	0.21*** (0.05)	0.39*** (0.04)	0.47*** (0.04)	0.31*** (0.04)
**Purpose in life**								
*R*^2^ adj.	–	0.01	0.14	0.32	0.21	0.34	0.44	0.27
*R*^2^ change	–	0.01	0.13	0.18	0.07	0.20	0.30	0.13
β (*SE*)	–	0.11*** (0.00)	0.36*** (0.03)	0.44*** (0.05)	0.27*** (0.05)	0.46*** (0.04)	0.60*** (0.04)	0.37*** (0.04)
**Personal growth**								
*R*^2^ adj.	–	0.01	0.04	0.16	0.09	0.12	0.15	0.18
*R*^2^ change	–	0.01	0.04	0.12	0.05	0.08	0.11	0.14
β (*SE*)	–	0.05* (0.00)	0.20*** (0.03)	0.36*** (0.04)	0.23*** (0.05)	0.29*** (0.04)	0.36*** (0.04)	0.39*** (0.04)
**Overall psychological well-being index**								
*R*^2^ adj.	–	0.01	0.21	0.44	0.28	0.38	0.44	0.35
*R*^2^ change	–	0.01	0.20	0.23	0.07	0.17	0.23	0.13
β (*SE*)	–	0.11*** (0.00)	0.45*** (0.02)	0.49*** (0.03)	0.28*** (0.03)	0.43*** (0.03)	0.52*** (0.03)	0.38*** (0.03)
**Somatization**								
*R*^2^ adj.	0.05	0.05	0.09	0.09	–	0.11	0.10	–
*R*^2^ change	0.05	0.01	0.04	0.00	–	0.02	0.01	–
β (*SE*)	0.22*** (0.03)	–0.08** (0.00)	–0.20*** (0.02)	–0.07* (0.03)	–	–0.15*** (0.03)	–0.11*** (0.03)	–
**Obsessive–compulsive**								
*R*^2^ adj.	0.01	0.04	0.14	0.18	0.15	0.23	0.18	0.17
*R*^2^ change	0.01	0.03	0.10	0.05	0.01	0.09	0.04	0.03
β (*SE*)	0.11***(0.04)	–0.18*** (0.00)	–0.31*** (0.02)	–0.22*** (0.04)	–0.12*** (0.04)	–0.31*** (0.04)	–0.22*** (0.04)	–0.17*** (0.04)
**Interpersonal sensitivity**								
*R*^2^ adj.	0.01	0.05	0.17	0.23	0.20	0.23	0.20	0.19
*R*^2^ change	0.01	0.04	0.13	0.06	0.03	0.06	0.03	0.02
β (*SE*)	0.09*** (0.03)	–0.20*** (0.00)	–0.36*** (0.02)	–0.24*** (0.03)	–0.18*** (0.03)	–0.25*** (0.03)	–0.19*** (0.03)	–0.15*** (0.03)
**Depression**								
*R*^2^ adj.	0.02	0.05	0.22	0.25	0.23	0.27	0.29	0.23
*R*^2^ change	0.02	0.03	0.17	0.04	0.01	0.06	0.07	0.02
β (*SE*)	0.16*** (0.04)	–0.16*** (0.00)	–0.41*** (0.02)	–0.21*** (0.04)	–0.12*** (0.04)	–0.25*** (0.04)	–0.30*** (0.04)	–0.15*** (0.04)
**Anxiety**								
*R*^2^ adj.	0.02	0.06	0.13	0.14	0.14	0.17	0.14	0.13
*R*^2^ change	0.02	0.04	0.07	0.02	0.01	0.04	0.02	0.01
β (*SE*)	0.14*** (0.03)	–0.20*** (0.00)	–0.26*** (0.02)	–0.14*** (0.03)	–0.11*** (0.03)	–0.22*** (0.03)	–0.15*** (0.03)	–0.09*** (0.03)
**Hostility**								
*R*^2^ adj.	–	0.04	0.10	0.12	0.14	0.15	0.12	0.12
*R*^2^ change	–	0.04	0.06	0.02	0.04	0.04	0.01	0.02
β (*SE*)	–	–0.21*** (0.00)	–0.25*** (0.02)	–0.13*** (0.04)	–0.19*** (0.03)	–0.22*** (0.03)	–0.13*** (0.03)	–0.13*** (0.03)
**Phobic anxiety**								
*R*^2^ adj.	–	0.01	0.05	0.06	0.06	0.07	0.06	0.05
*R*^2^ change	–	0.01	0.04	0.01	0.01	0.02	0.01	0.01
β (*SE*)	–	–0.09** (0.00)	–0.21*** (0.01)	–0.12*** (0.02)	–0.09** (0.02)	–0.14*** (0.02)	–0.12*** (0.02)	–0.08** (0.02)
**Paranoid ideation**								
*R*^2^ adj.	–	0.04	0.16	–	0.18	0.17	0.16	–
*R*^2^ change	–	0.04	0.12	–	0.02	0.02	0.00	–
β (*SE*)	–	–0.20*** (0.00)	–0.35*** (0.02)	–	–0.14*** (0.04)	–0.13*** (0.04)	–0.06* (0.04)	–
**Psychoticism**								
*R*^2^ adj.	–	0.03	0.15	0.16	0.16	0.19	0.17	0.16
*R*^2^ change	–	0.03	0.12	0.01	0.01	0.04	0.02	0.01
β (*SE*)	–	–0.19*** (0.00)	–0.34*** (0.02)	–0.12*** (0.03)	–0.12*** (0.03)	–0.20*** (0.02)	–0.14*** (0.02)	–0.11*** (0.02)
**Global Severity Index (GSI)**								
*R*^2^ adj.	0.02	0.05	0.19	0.22	0.21	0.26	0.23	0.21
*R*^2^ change	0.02	0.04	0.14	0.03	0.02	0.07	0.04	0.02
β (*SE*)	0.13*** (0.03)	–0.20*** (0.00)	–0.37*** (0.02)	–0.18*** (0.03)	–0.15*** (0.03)	–0.27*** (0.03)	–0.21*** (0.03)	–0.14*** (0.03)

*The entries are adjusted *R*^2^ (*R^2^ adj.*) for the regression model, *R*^2^ changes at each step of the regression equation, standardized (β) regression coefficients obtained when the variable was first entered, and *SE* (standard error).*

*p < 0.05; **p < 0.01; ***p < 0.001; (a) 1- Man, 2- Women.

In the current sample, the sociodemographic characteristic of age was a significant predictor for all components of PWB except for the dimension of positive relations with others. In contrast, gender only entered the model for this dimension of positive relations with others (β = 0.14) at a significant level in step 1. Again, the value of *R*^2^ increased significantly when functional social support entered all models. This predictor produced the highest increment of *R*^2^ for the dimension of positive relations with others (*R*^2^ change = 0.23), and the lowest significant increment of this value for the component of autonomy (*R*^2^ change = 0.02). Regarding character strengths, theological strengths made the strongest contribution to the variance explained for the dimensions of purpose in life (β = 0.60), self-acceptance (β = 0.50), environmental mastery (β = 0.47), and positive relations with others (β = 0.25). For autonomy, the predictor variable of emotional strengths made the strongest contribution to the variance explained (β = 0.41). For the dimension of personal growth, the factor of intellectual strengths was the strongest predictor (β = 0.39).

In this sample, age was a significant predictor for all symptoms of psychopathology. Specifically, lower age predicted higher level of psychopathology, with standardized (β) regression coefficients ranging from –0.08 (somatization) through –0.21 (hostility). In addition to age, the sociodemographic characteristic of gender was a significant predictor for somatization (β = 0.22), depression (β = 0.16), anxiety (β = 0.14), obsessive-compulsive symptoms (β = 0.11), and interpersonal sensitivity (β = 0.09). In the third step, functional social support was found to be a variable with predictive value in explaining the variability of each dimension of psychopathology. This predictor variable produced the highest increment of *R*^2^ for depression (*R*^2^ change = 0.17), and the lowest significant increment of this value for somatization and phobic anxiety (*R*^2^ change = 0.04). Concerning character strengths as predictors of psychological problems, results showed that strengths of restraint had the greatest predictive power for the Global Severity Index (β = –0.27) and all subscales with the exceptions of the subscales of depression and paranoid ideation. In particular, standardized (β) coefficients ranged from –0.14 (phobic anxiety) through –0.31 (obsessive–compulsive symptoms). For depression, the factor of theological strengths made the strongest contribution to the variance explained (β = –0.30). For the subscale of paranoid ideation, the factor of interpersonal strengths was found to be the most potent predictor (β = –0.14) in the final model.

## Discussion and Conclusion

The present study examines the incremental effects of strength factors over and above functional social support on SWB, PWB, and psychopathological symptoms in Spanish university students. As in previous investigation (e.g., Ferreira and Sherman, 2007; Skomorowsky, 2014), we found that functional social support was significantly associated with the components of SWB and the dimensions of PWB, mainly to life satisfaction and positive relations with others. Additionally, the results indicated a significant negative relationship between functional social support and symptoms of psychopathology, especially depression. These findings certainly support the previous research (e.g., Southwick et al., 2005; Costa et al., 2012), suggesting that functional social support could protect people from psychological problems by buffering the negative effect of life stressors on mental health and promoting well-being.

The comparison of the predictive capacity of functional social support and character strengths revealed that the contribution of functional social support to the explanation of the variability of the global indicator of psychological distress (i.e., GSI) was greater than the contribution made by character strengths. However, for the SWB and PWB indicators, the character strengths tended to have greater predictive power than functional social support. Therefore, although both character strengths and functional social support are resources significantly associated with higher degrees of psychological health and lower levels of psychiatric disorders, our results indicate that the importance of each of them is different in the prediction of the mental health indicators examined.

In line with most of the previous evidence (e.g., Park et al., 2004; Leontopoulou and Triliva, 2012; Buschor et al., 2013; Proyer et al., 2013b; Weber et al., 2013), the present study showed significant associations between the five strength factors and all components or dimensions of SWB and PWB. Specifically, theological strengths (i.e., spirituality, gratitude, zest, hope, and love) were the strongest predictive variables of life satisfaction, positive affect, affect balance, environmental mastery, purpose in life, and self-acceptance. This group of strengths provides meaning to our lives (Peterson and Seligman, 2004). Thus, considering oneself a spiritual person and finding meaning in everyday life improves our overall sense of well-being, as it helps to give purpose to one’s life and increases optimism. Likewise, gratitude is one of the strengths most closely related to experiencing a meaningful life; being aware of and grateful for the good things that happen leads to experiencing a variety of positive emotions. Similarly, zest is one of the character strengths most strongly connected to well-being because it involves feeling vital and enthusiastic, and approaching life with full energy. The character strength of hope involves having optimistic thoughts and positive expectations about the future, as well as focusing attention on the good things to come. This probably explains why hope is one of the strengths most associated with life satisfaction and general well-being. Moreover, love tends to facilitate empathy and forgiveness in relationships, which contributes to strengthening them. Experiencing close relationships characterized by giving and receiving love is strongly associated with greater life satisfaction and positive emotions.

In addition, theological strengths were the most relevant character strengths factor for the dimension of positive relations with others, though interpersonal strengths (i.e., fairness, kindness, teamwork, modesty, and forgiveness) and emotional strengths (i.e., social intelligence, leadership, perspective, creativity, bravery, and humor) made also similar contributions to this dimension. What could be the reason for this? The interpersonal strength of fairness helps to promote positive relationships with others because it facilitates the manifestation of positive and prosocial behaviors and, at the same time, decreases the likelihood of behaving immorally. Kindness involves being caring, loving, compassionate and helpful to other people; this concern for the well-being of others could create opportunities to build meaningful relationships with them. Similarly, teamwork leads to a higher level of social trust and a more favorable perception of others. Additionally, being modest, not seeking the limelight and not considering oneself more important than others can facilitate the maintenance of quality relationships. Humble people tend to show higher levels of forgiveness, which in turn can help foster the development of constructive interpersonal relationships. Social intelligence, i.e., the ability to understand the feelings and thoughts of both oneself and others, has been associated with better social relationships and mental health. The character strength of leadership is related to social intelligence. Scoring high on leadership is associated with greater respect and esteem from others. In the same way, people with perspective and creativity are valued by others because they can see what is best for the situation or problem being discussed and have a facility for thinking of many ways to solve a problem; this enables them to give good advice and assist in decision making. Likewise, bravery and humor can help create opportunities for social connection and for developing and maintaining close relationships. Bravery helps people tolerate the vulnerability that is part of approaching others, while humor reduces social anxiety when we interact with them.

Moreover, intellectual strengths (i.e., love of learning, curiosity, and appreciation of beauty and excellence) were the most important strengths in promoting the dimension of PWB labeled personal growth. Love of learning and curiosity are closely related strengths. Both character strengths are associated with healthy, productive aging. Being curious implies having a strong desire to increase one’s personal knowledge and being open to experience, whereas love of learning refers to be highly motivated to expand on existing knowledge in a meaningful way. The pursuit of new activities and experiences contributes to the dimension of personal growth. Likewise, a passion for learning contributes to a sense of continuous personal development by deepening knowledge, enhancing personal competencies and skills, and helping to persist through challenges and obstacles. The third intellectual strength is the appreciation of beauty and excellence, which refers to recognizing, emotionally experiencing, and appreciating the beauty that surrounds us and the ability of others. People with a high level of this character strength notice and appreciate beauty and excellence in all areas of life. Appreciating, for example, a skill or talent (excellence) can be energizing and promote the person to pursue his or her own goals and thus achieve greater personal growth.

Finally, we found that character strengths were substantially related to fewer symptoms of psychopathology. These findings support previous research (e.g., Gander et al., 2013). Strengths of restraint (i.e., self-regulation, prudence, persistence, open-mindedness, and honesty) seemed to have the highest protective power for most of the psychological problems, specifically for obsessive-compulsive symptoms, interpersonal sensitivity, anxiety, hostility, psychoticism, somatization, and phobic anxiety. However, depressive symptoms were best explained by theological strengths; this is probably because the feelings of hopelessness and loss of vital energy that characterize depression are the opposite of the character strengths of hope and zest.

Why do people with high scores on the strengths of restraint report fewer psychopathological symptoms? First, self-regulation refers to self-control and management of emotions and behaviors; it is related to optimal psychological functioning and personal adjustment. The character strength of prudence refers to being careful, acting with caution, thinking before acting, not taking unnecessary risks, analyzing the possible consequences of one’s actions, and controlling oneself based on this analysis. This strength of restraint helps to avoid mishaps, setbacks or problems in life that can lead to psychological suffering. Moreover, the strength of persistence involves voluntarily continuing an action to achieve a goal despite the presence of obstacles or difficulties along the way. Being persistent and achieving one’s goals leads to increased self-confidence and self-esteem; in addition, perseverance can help develop other character strengths. Additionally, open-mindedness involves seeing things from more than one perspective, weighing all aspects objectively when making decisions, being able to change one’s mind considering evidence, and remaining open to other ideas and perspectives. People who score high in open-mindedness are especially capable of coping with periods or times of change and uncertainty. Lastly, honesty refers to being consistent with oneself, being a person of integrity, being who you say you are and acting accordingly. Honest people are people who can be trusted, which facilitates the development of healthy and positive relationships with others. Therefore, all strengths of restraint involve traits and capacities that contribute, in different ways, to maintaining optimal psychological functioning and preventing the onset of psychopathological symptoms.

Overall, the present results support most of the growing empirical evidence regarding the correlates and outcomes of character strengths. Our findings show that character strengths are positively associated with components of SWB and PWB, and negatively associated with psychological distress. This could be interpreted as support for the assumption that character strengths are important resources for mental health, by promoting well-being and preventing psychological disorders. The five higher-order strength factors are significantly associated with well-being and mental health, though certain strength factors are more robustly linked to specific outcomes than others. Thus, in general terms, theological strengths (e.g., hope, zest, gratitude) appear to be the most beneficial for subjective and PWB, while strengths of restraint (e.g., self-regulation, prudence, persistence) are those that would contribute most to preventing psychological distress.

On this basis, those character strengths that contribute greatly to lower levels of psychopathology and higher levels of well-being should be prioritized as targets in strength-based interventions. Knowing this evidence could be helpful in the design and development of effective interventions to enhance mental health in various contexts, not only in the clinical setting. In accordance with this, the approach of *positive psychotherapy* (PPT; Seligman et al., 2006), which emerged from the field of positive psychology, seems to offer an effective way to treat and prevent psychopathology, specifically depression. According to PPT, developing character strengths, positive emotions, and meaning may counteract negative symptoms and even buffer against their future reoccurrence. However, when we think about these positive psychology interventions, we must refer to the so-called “signature strengths” (Peterson and Seligman, 2004). Identifying and fostering those character strengths that are most representative of a person should be also a fundamental part of interventions within this area of PPT.

In general, there is robust evidence that interventions targeting signature strengths are effective in raising various indicators of well-being; results regarding depression are mixed, but they point to a potential contribution for ameliorating depressive symptoms (Proyer et al., 2015). At the same time, research has also shown that it is fruitful to work on those character strengths most associated with the components of well-being (e.g., Proyer et al., 2013b; Graziosi et al., 2020; Yan et al., 2020). Given this evidence, the question that arises is whether it would be more beneficial for mental health to design interventions targeting signature strengths or interventions targeting the strengths that we found in our study to be more strongly associated with mental health (i.e., theological strengths and strengths of restraint, primarily) even in the case where neither of these had been identified as signature strengths. Another question that emerges in this regard is whether it would be more beneficial that interventions be designed individually according to the levels of indicators of well-being and psychopathology. Answering these questions remains a challenge for future research.

The present study involves the collection of data from a unique sample at a particular time point. Most of evidence that analyzes the role of social support or character strengths on well-being also derives from cross-sectional studies. Longitudinal designs are needed in order to test our findings and confirm the predictive relationship assumed from the theoretical model about the role of functional social support and character strengths on well-being. Furthermore, participants were recruited from Spanish universities. For better generalizability, future research should collect data from different contexts. Further works with clinical samples are suggested to analyze the role of functional social support and character strengths in different psychological and psychiatric disorders. Additionally, the measures used in this study were based exclusively on self-report questionnaires, in which social desirability bias could be of particular concern. This limitation indicates that future studies should be conducted to further develop multimethod strategies to assess character strengths and functional social support and to validate self-report data.

To conclude, functional social support and character strengths seem to be positive resources that have important consequences for well-being. The design of positive interventions to cultivate these personal and social resources will help us experience fewer psychological problems and improve our state of mental health and well-being. Future research should continue to identify the mechanisms of action on mental health underlying each of these resources. Working on character strengths is effective in promoting well-being, but further research is needed on how these interventions could be tailored to the individual to maximize their effectiveness.

## Data Availability Statement

The raw data supporting the conclusions of this article will be made available by the authors, without undue reservation.

## Ethics Statement

The studies involving human participants were reviewed and approved by the present study protocol was authorized by the Ethics Committee of the Villanueva University (Madrid, Spain), with the reference 2020-27. The ethics committee waived the requirement of written informed consent for participation.

## Author Contributions

CA and SS: investigation. JA: methodology. TA: supervision. CA: writing original draft. All authors read and agreed to the published version of the manuscript.

## Conflict of Interest

The authors declare that the research was conducted in the absence of any commercial or financial relationships that could be construed as a potential conflict of interest.

## Publisher’s Note

All claims expressed in this article are solely those of the authors and do not necessarily represent those of their affiliated organizations, or those of the publisher, the editors and the reviewers. Any product that may be evaluated in this article, or claim that may be made by its manufacturer, is not guaranteed or endorsed by the publisher.
